# Microstructure and In Vitro Evaluation of Extruded and Hot Drawn Alloy MgCa0.7 for Biodegradable Surgical Wires

**DOI:** 10.3390/ma14216673

**Published:** 2021-11-05

**Authors:** Andrij Milenin, Mirosław Wróbel, Piotr Kustra, Dorota Byrska-Wójcik, Joanna Sulej-Chojnacka, Bartłomiej Płonka, Krzysztof Łukowicz, Karolina Truchan, Anna Osyczka

**Affiliations:** 1 Faculty of Metals Engineering and Industrial Computer Science, AGH University of Science and Technology, al. Mickiewicza 30, 30-059 Kraków, Poland; mwrobel@agh.edu.pl (M.W.); pkustra@agh.edu.pl (P.K.); byrska@agh.edu.pl (D.B.-W.); 2 Łukasiewicz Research Network—Metal Forming Institute, ul. Jana Pawła II 14, 61-139 Poznań, Poland; joanna.sulej-chojnacka@inop.lukasiewicz.gov.pl; 3 Division in Skawina ul. Piłsudskiego 19, Lukasiewicz Research Network—Institute of Non-Ferrous Metals, 32-050 Skawina, Poland; bplonka@imn.skawina.pl; 4 Department of Cell Biology and Imaging, Institute of Zoology and Biomedical Research, Faculty of Biology, Jagiellonian University, Gronostajowa 9, 30-387 Kraków, Poland; krzysztof.lukowicz@doctoral.uj.edu.pl (K.Ł.); karolina.truchan@doctoral.uj.edu.pl (K.T.); anna.osyczka@uj.edu.pl (A.O.)

**Keywords:** biodegradable surgical wire, magnesium alloys, in-vitro bio-corrosion, cytotoxicity, anti-tumour activity

## Abstract

The MgCa0.7 alloy may be a promising material for biodegradable surgical wires. In this paper, the technology for producing surgical wires from this alloy has been developed, based both on finite element modelling and experimental study. In particular, the extrusion and hot-drawing effects on the mechanical properties, microstructures, in-vitro rates of biocorrosion, and cytotoxicity to human cancer cells (SaOS-2) and healthy (hPDL) ones, have been determined. An approximately 30–40% increase in corrosion rate due to increasing hot-drawing temperature was observed. An effect of hot-drawing temperature on cytotoxicity was also found. Notably, at various stages of the final wires’ production, the MgCa0.7 alloy became toxic to cancer cells. This cytotoxicity depended on the alloys’ processing parameters and was maximal for the as-extruded rod and for the wires immediately after hot drawing at 440 °C. Thus, the careful selection of processing parameters makes it possible to obtain a product that is not only a promising candidate for biodegradable surgical wires, but one which also has intrinsic bioactive properties that produce antitumor activity.

## 1. Introduction

Biodegradable surgical wires (BSW) with high strength are still in demand for surgical practice. Today, metallic materials are regaining the attention of medical engineers for such application. Magnesium-based alloys are one of the best candidates for BSW manufacturing [1]. The main advantage of magnesium is its good biocompatibility. The recommended daily intake of Mg for the human body is between 375 and 500 mg/day [2]. For comparison, according to [3], the recommended daily intake of iron varies between 6 and 20 mg/day and for zinc between 6.5 and 15 mg/day [4]. According to [5], pure magnesium wires were used in surgery for the first time by M.D. Edward and C. Huse in 1878. Huse successfully used them as ligatures to stop bleeding vessels in a radial artery and during an operation for varicocele. In conclusion of the clinical report, Huse wrote the following:

“It has seemed to me that this ligature will be of a special value in ovariotomy, where it is desirable to tie the vessels of the pedicle and return it into the abdomen, and in operations for hemorrhoids. It can be used everywhere under all conceivable circumstances and contingencies; it will never break; it is always ready; it cannot untwist like cat gut or silk; it can neither slip, become stiff or rotten; it can never provoke irritation, absorb moisture, disappoint or cause anxiety, but will always act, “tuto cito et jucunde”. It seems to me, finally, that it must supersede all ligatures, because it is not only better, safer, more convenient and needful, but the only thing necessary to overcome the whole category of objections on either one of which, lives of priceless value have in host of causes been dependent”.

Broader clinical studies of magnesium wires started around the end of the first half of the 20th century, after the advent of large-scale magnesium production technology [6]. Magnesium was used for wound closures and the fixture of fractured bones, for the anastomosis of blood vessel and intestines, as ligatures for blood vessels and as a surgical suture (e.g., [7,8]). During this period of time two important observations were made. The first work indicated the corrosive biodegradation of magnesium, in vivo [8]. Following this, several problems associated with the use of magnesium wires in the mammalian body were revealed, such as its intensive hydrogen gas release during bio-corrosion, as it cannot be metabolized in some cases and so forms cavities that impede healing. Due to the developments of several alternative materials in the second half of the 20th century, the latter issue contributed to gradual decline in the interest in using metallic magnesium as a surgical biomaterial. However, research done in the early 21st century revealed that the corrosion rate of magnesium can be significantly reduced by alloying (e.g., by the addition of calcium [9]) and/or by coating [10]) and the interest in using magnesium in surgery was revived.

Recent studies suggest that the rate of biocorrosion of magnesium alloys depends on many factors, including its microstructure and surface roughness. For example, in the work [11] the extruded alloy Mg-1Ca-0.5Zr showed a lower rate of corrosion after heat treatment and a higher one with an increased roughness of the sample surface. Unfortunately, the parameters of extrusion and the quantitative effect of the heat treatment on the corrosion are not given. The obtained values of the corrosion rate in the R-SBF environment on the third day of immersion were 2 mm/year for the heat-treated material and about 60 mm / year for the material with artificially increased roughness. 

The effect of heat treatment on the rate of biocorrosion of wire made of the Resole alloy (Mg-10Dy-1Nd-1Zn-0.2Zr) was studied in [12]. Wires with a diameter of 0.5 and 0.2 mm were obtained by cold drawing, with various subsequent heat treatments. Immersion results in M-SBF environment showed that the wire exhibited relatively uniform corrosion with average rates of 1.72 ± 0.09, 1.44 ± 0.21, and 1.97 ± 0.10 mm/year for cold-deformed wires and those annealed at 350 °C and 450 °C, respectively.

To sum up, the effects of heat treatment were not uniform, depending on the annealing temperature, but were insignificant in magnitude. On the first day of immersion, the corrosion rate was much higher than the average value and ranged from 35 mm/year to 15 mm/year.

In-vitro and in-vivo studies of the Mg1Ca alloy in an as-cast state were carried out in [13]. The average rate of biocorrosion in the SBF solution was 21.01 mm/year. In Dulbecco medium, this value was significantly lower, 3.45 mm/year. The corrosion rate of the same material, in vivo, was even lower and was 1.34 in bones and 0.7 in muscles. Thus, the in-vitro corrosion rate can be used as a relative assessment of the effects of different factors on overall corrosion rate but has little correspondence with the absolute values of the in-vivo corrosion rate. Cytotoxicity studies in this work were performed on the SaOS-2 cell line, at an extract concentration of 0.2 g/mL. The results with an incubation time of 24 h showed high cell survival (above 70%), while incubation for 72 h led to slightly worse results (30.8–70.4%). In general, this work once again confirmed the promising nature of using MgCa alloys as a basis for implants. However, the effects of the material’s processing and its microstructure on its biocorrosion and cytotoxicity remain to be elucidated. 

The effect of rolling on the rate of biocorrosion and cytotoxicity was studied in [14] for samples of MgZn0.7Ca0.6 alloy obtained through twin-roll casting. The biocorrosion rate was investigated in modified Eagle medium alpha (α-MEM) with 10% foetal bovine serum (FBS). It was found that, on the third day of immersion, the corrosion rate of the rolled material was 0.17 mm/year, and that of the annealed material after rolling was 0.1 mm/year. The obtained values, firstly, are the lowest values of all the analysed results, and secondly, they indicate a decrease in the corrosion rate after annealing.

Overall, the available results on in-vitro corrosion rates do not agree with each other and strongly depend on the medium used and the composition of the alloy and its processing. There is no reliable data on the effect of hot or cold metal-forming parameters on biocorrosion, which is extremely important when developing a technology for the production of surgical wires. 

Different magnesium alloys are currently proposed for soluble implants [15] and tested for BSW. A magnesium alloy with low calcium content has been selected as the subject of the current study. According to [9], such alloys are promising candidates for BSW. Indeed, calcium significantly reduces the corrosion rate of magnesium, and its minimum corrosion rate if obtains when calcium content is 0.6% to 0.8%, as reported by [10]. On the other hand, according to [16], an increase in the calcium content to 2% not only reduces the alloy’s mechanical properties (i.e., both the strength and plasticity) but also deteriorates the corrosion resistance. A decrease in the in-vitro bio-corrosion resistance of magnesium due to an increase in the Ca content from 0.9% to 1.2% was recently confirmed by us [17]. Thus, based on the available data, it is plausible to consider the alloy composition, selected in the present study, as optimal. In our previous report, the results regarding the bio-corrosion of the extruded Mg-Ca rods with a diameter of 1.8 mm were presented [18]. The manufacturing of a smaller-diameter wire requires using a drawing process. However, conventional wire-drawing of Mg-Ca alloys is relatively difficult. The main limiting factor is the low workability of the Mg-Ca alloys at room temperature and very low ductility of the final wire [19]. For this reason, to obtain a wire suitable for surgical knots, a multi-pass hot-drawing process should be employed [20]. However, a hot-drawing schedule (i.e., the distribution of partial reductions in the area, drawing velocity and temperature) must be chosen carefully, especially in the case of such difficult metals as Mg-Ca alloys. An error can lead to the intermediate/final wire embrittlement. The development of the hot drawing processing maps requires the control of material microstructure. The effect of microstructure on the bio-corrosion of magnesium alloys has also been reported [21,22,23,24]. On the other hand, hot-drawing allows the microstructure to be influenced by the temperature of the deformation and by the strain rate. This opens up the prospect of controlling the microstructure and, consequently, the rate of biocorrosion and cytotoxicity. Evidence for the effect of processing route and microstructure on the cytotoxicity of magnesium alloys can be found in the literature, e.g., [25]. In this work, the effect of equal channel angular pressing on the in-vitro anti-cancer activity of the magnesium alloy WE43 (Mg—balance, Y—3.56%, Nd—2.20%, Zr—0.47%) has been examined. However, the limitation of this work is that the effect of our processing parameters on the cytotoxicity of healthy cells has not been considered. The cytotoxicity of MgCa0.6, MgCa1.0 and pure-Mg alloys to cancer (osteosarcoma cell line MG63) and healthy (osteoblasts obtained from two patients) cells was studied by [26]. Cast material was used for these experiments and it was found that the alloys MgCa0.6 and MgCa1.0 are highly toxic to cancer cells but do not display any toxicity to healthy cells. However, this cannot be simply translated to an extruded and/or hot-drawn wire with a completely different microstructure. In the present work, we have assumed that not only the mechanical properties but also the bio-corrosion rate and cytotoxicity of magnesium alloys depend on the final product’s microstructure, which, to some extent, can be controlled by the hot-drawing temperature. To the best of our knowledge, such combined microstructure effects have been overlooked regarding bio-magnesium alloys. In particular, we have not found any data on the hot-drawing parameters’ consolidated effects on Mg-Ca alloys’ microstructures, properties, bio-corrosion rates or cytotoxicity. 

The development of wire-processing technology for the MgCa0.7 alloy for BSW and the specification of the microstructural parameters relevant for this application were the main aims of this study. This goal has been achieved through a combination of finite element (FE) computer modelling and an experimental study. Additionally, the effect of extrusion and hot drawing on the anti-cancer activity of the final product has been examined.

## 2. Materials

### 2.1. Alloy Production

Mg-30 wt. %Ca master alloy and commercial-purity magnesium were used for the alloy MgCa0.7 ingots’ preparation. The metals were melted in a Hindenlang resistance furnace and cast from a temperature of 690–710 °C in a HOT-TOP crystallizer with a diameter of 102 mm. The obtained billet was a multi-channel die extruded and then hot-drawn according to the deformation schedule determined by FE modelling, presented in Section 3. The obtained alloy had the following chemical composition (in wt. %): Ca—0.70; Zn—0.0015; Mn—0.01753; Pb—0.00112; Al—0.02646; Fe—0.00269; Si—0.01699; Acceptable admixtures—0.29; Mg—balance.

### 2.2. Flow Stress

Plastometric compression tests of the alloy were carried out on a Gleeble 3800-GTC (Dynamic Systems Inc., Poestenkill, NY, USA). Cylindrical samples with an initial diameter of 8 mm and a height of 10 mm were used. The ranges of strain rate and temperature were 0.01–1 s^−1^ and 250–400 °C, respectively. During tests, the temperature was controlled by thermocouples attached to the sample. A detailed description of the method and the results of the flow-stress measurements are presented elsewhere [18].

### 2.3. Models of the Recrystallization

To predict the microstructure of the extruded and hot-drawn material an integration of the recrystallization model into the finite element model (FEM) of the deformation process was needed. Recrystallization can be treated as a solid-state phase transformation. An equation describing the kinetics of such an isothermal transformation was derived by Kolgmogorov [27] and Johnson and Mehl [28], popularized by Avrami [29], and adapted to dynamic recrystallization by Sellars [30]. The phase-transformation model and the corresponding equation are known in the literature as the JMAK model/equation. The JMAK model of static recrystallization can be expressed in an incremental formulation by the following equations:

(1) $$X_{stat}=1-\exp\left(c_{5}{\left(\frac{\tau }{\tau_{0.5}}\right)}^{n}\right),$$

(2) $$\tau_{0.5}=c_{1}\varepsilon^{c_{2}}\exp\left(c_{3}\varepsilon \right)d_{0}\exp\left(\frac{c_{4}}{RT}\right),$$

(3) $$\tau_{0.95}=\tau_{0.5}\frac{\ln\left(0.05\right)}{c_{5}},$$

(4) $$∆X_{stat}=\frac{∆\tau }{\tau_{0.95}},$$

where *X_stat_* is the static recrystallized volume fraction, *τ_0.95_* and *τ_0.5_* is the isothermal annealing time for *X_stat_* = 95% and *X_stat_* = 50%, respectively, *T* is the absolute temperature, *ε* is the true strain before recrystallization, *d*_0_ is the initial grain size, and *c*_1_-*c*_5_ and *n* are material constants that can be fit. The values of the material constants in Equations (1)–(4) were determined for MgCa0.7 alloy and presented in our previous paper [18], are: *c*_1_ = 3.780 × 10^−7^; *c*_2_ = −0.4081; *c*_3_ = 5.356; *c*_4_ = 45976.4; *c*_5_ = −0.6797; *n* = 0.5053. 

In the present paper, it has been assumed that static recrystallization occurs mainly during hot drawing, thus the static recrystallization model, only, was used for this process. 

During the extrusion process of the rods—the hot-drawn workpiece—the recrystallization can occur. Since dynamic recrystallization may prove important during extrusion, the JMAK model of dynamic recrystallization was applied. The model can be expressed by the equations:

(5) $$\varepsilon_{c}=Ad_{0}^{p}Z^{q}$$

(6) $$Z=\;\dot{\varepsilon }\;\exp\left(\frac{Q}{RT}\right)$$

(7) $$X_{dyn}=1-\exp\left[-C{\left(\frac{\varepsilon -\varepsilon_{c}}{\varepsilon_{s}-\varepsilon_{c}}\right)}^{2}\right]$$

where *X_dyn_* is the dynamically recrystallized volume fraction, *ε* and *ε_c_* are the actual strain and the critical strain for dynamic recrystallization, *ε_s_*, *A*, *p*, *q*, and *C* are material constants, *Z* is the Zener–Hollomon parameter, $\dot{\varepsilon }$ is the strain rate, *Q* is the activation energy, *R* is the gas constant, and T is the deformation temperature, in Kelvin. The methodology for the above parameters determination based on the results of the plastometric test was described elsewhere [31]. In this work, the material constants values in Equations (5)–(7) were determined (from the maximum value in the strain - flow stress curves) to be: *A* = 0.0377, *p* = 0.4, *q* = 0.06945, *ε_s_* = 0.8, *C* = 3, *Q* = 85,000 J/mol.

## 3. Method

### 3.1. Extrusion 

The cast billets were extruded using a multichannel die into rods with a diameter of 1.8 mm. The deformation zone’s temperature was 460 °C and the extrusion velocity was 0.25 mm/s. A hydraulic press, with a maximum force of 5 MN, was used. The effective strain of the material during extrusion reached 6.0–7.0. Detailed information about the extrusion process was present by [17]. After extrusion, the material was hot drawn. 

### 3.2. Hot Drawing 

**Figure 1:**
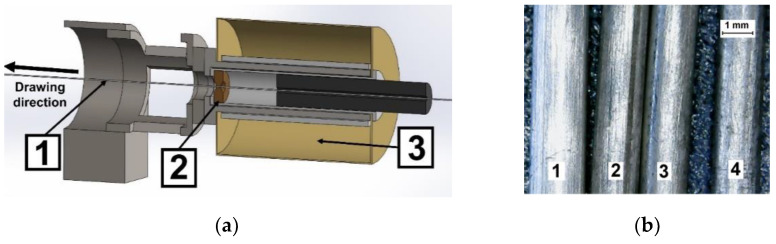
Technology of wire production: (**a**) hot-drawing processes used in the experiment: 1—wire; 2—drawing die; 3—heating device; (**b**) wires, obtained by extrusion (1), hot drawing in temperature conditions t350 (2), t400 (3), and t440 (4).

The idea of hot drawing can be illustrated in Figure 1. A cold billet (extruded rods with a diameter of 1.8 mm and a length of 2–5 m) was unwound from a coil and heated while being passed through a specially designed furnace (no. 3 in Figure 1) into a heated die (no. 2 in Figure 1). The die was mounted inside the furnace; therefore, the material was deformed at an increased well-controlled temperature. After passing through the deformation zone, the wire was coiled by the engine and cooled to room temperature. In such a process, the wire’s drawing velocity affects not only its strain rate, but also its deformation temperature. Increase in the drawing velocity decreases the heating time of the wire and lowers the deformation temperature. On the other hand, reducing wire diameter accelerates heating. Thus, the determination of the deformation temperature, in such process, is a nontrivial task, requiring FEM modelling. This concept of the deformation process was tested in our previous studies, and a more detailed description has been published (e.g., [20]).

The hot drawing velocity is precisely controlled by special software regulating the coiler’s driving engine. At the end of the passage, the coils were replaced and the process was repeated. The drawing schedule for three passes is presented in Table 1.

**Figure 2:**
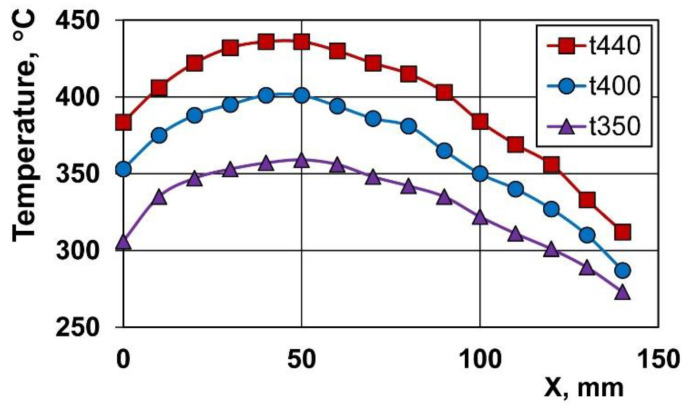
Temperature distribution of the wire in the furnace. Drawing direction—right to left.

The furnace temperature was determined in separate experiments and the temperature distribution along the furnace length (Figure 2) was measured. In this figure, the drawing direction is from right to left, as in Figure 1, so the original temperature (for X = 0) corresponds to the die temperature. Three temperature distributions in the furnace shown in Figure 2 (called as t350, t400, and t440) were used for the computer simulations and experiments selected to this paper. The drawing die angle was 5°, a graphite-based lubricant was used, and the drawing velocity was 10 mm/s. Afterward, the drawn wires were first cleaned with soap and water, then, ultrasonically, in ethanol, and finally dried at room temperature. 

### 3.3. FEM Simulations

The estimation of the conditions of the deformation during the extrusion and hot drawing was a goal of the FEM simulations. A rigid-plastic non-isothermal FE model has been applied. Qform *VX* (a commercial FE software (ver 9.0, Micas Simulations Limited, Oxford, United Kingdom)) was used for the simulation, similarly as in Biba et al. [32]. In FE codes, recrystallization models were implemented as subroutines written in the programming language LUA. For the alloy MgCa0.7, it can be assumed that *c* = 1013.4 + 0.441*t*, *ρ* = 1741.4 − 0.173*t* and *λ* = 156.32 − 0.023*t*, where *c,* *ρ,* and *λ* are, respectively, the alloy-specific heat, density, and thermal conductivity coefficient, and *t* is the temperature in °C [33]. Numerical data of flow stress–strain curves, in the form of tabular functions, were used during the simulation.

### 3.4. Mechanical Testing

A Zwick250 (Zwick Roell, Ulm, Germany) tensile testing machine equipped with specially designed grips, was used for the tensile tests (see [34], Figure 5). For all samples, the ratio of a sample’s gage length to its diameter was the same as and not less than 200, and the tensile rate was 20 mm/min. The requirements of ISO 6892-1:2019 were met. The wires’ bending tests, assessing their suitability for knotting, was quantified by the reverse-bend-test results. Bending susceptibility (an important indicator for the assessment of product suitability for knotting) was quantified by the reverse bend test performed on a roll with a diameter of 2 mm. Recommendations of the standard A321 were followed.

### 3.5. Microstructure and X-ray Diffraction

A metallographic microscope Zeiss Axio Imager M1m (Zeiss, Oberkochen, Germany) was used for the microstructure studies carried out mainly on the wires’ longitudinal sections. Magnesium usually is covered by a surface film which can significantly influence corrosion [35]. Therefore, from the bio-corrosion point of view, the surface and subsurface layers of the material may be relevant. Therefore, X-ray diffraction (XRD) from such layers was measured. Thus, the cylindrical surface of the wire (i.e., parallel to the extrusion/drawing direction) was exposed to Cu-Kα radiation. An Empyrean diffractometer, from PANalytical (Malvern Panalytical B.V., Almelo, The Netherlands), was used. The data were collected during continuous scan, carried out in reflective mode, and applying parallel beam geometry (a Göbel mirror in the incident beam optics and a parallel plate collimator in the diffracted beam). The symmetrical diffraction was applied for the phase analysis measurement performed for the 2*Θ* angle range of 20–145 °C, step 0.02°. The Schultz back-reflection technique, described by [36], was used to determine crystallographic textures. The incomplete pole figures of the lattice planes: (00.2), (10.0), (10.1), (10.2), and (11.0) were measured on an equiangular measurement grid. The range of the polar angle (*α*’) and the azimuthal angle (*β*’) was of 0–75 °C and of 0–360 °C, respectively. The angle step was Δ *α*’ = Δ *β*’ = 5°. The orientation distribution function (ODF) and complete pole figures were calculated from the incomplete pole figures with the LaboTex software [37].

### 3.6. Bio-Corrosion

The in-vitro test was done in a mixture of bovine serum supplied by Biowest (catalog No. S0250 [38]). Protein content in the mixture was equal to 30 g/L. Sodium azide (0.3 wt.%) and 20 mM of ethylenediaminetetraacetic acid were added to the solution to inhibit bacterial growth and to bind calcium ions. The mixture was filtered through a sterile filter (pore size of 20 µm). During corrosion tests, the temperature of the corrosion medium mas maintained at 37 ± 0.1 °C. pH values were measured daily with a laboratory digital pH-meter. The pH value increased, to 8.0–8.2, during the first 10 days of the test, but subsequent days did not show such large changes in pH. During the last days of the test, changes in the pH value, in the range of 7.4–7.6, were observed. The protein concentration was monitored with a GenesysTM^20^ spectrophotometer (Spectronic Instruments Inc., Rochester, NY, USA). During the test, all recommendations given by the ASTM F732-00 standard were met. After each test period, the samples were removed from the corrosion medium and a change in their weight was determined using a laboratory balance with an accuracy of 0.00001 g. Weighing was carried out after 3, 7, and 14 immersion days. Immediately before the test and before each weighing, samples were thoroughly cleaned, first with the distilled water and then ultrasonically, in ethanol, and finally dried at room temperature according to the ASTM G1-03-E standard. 

The corrosion rate (in mm/year) was calculated from the equation:

(8) $$CR=365\frac{m_{0}-m_{corr}}{\rho \;A\;\tau },$$

where *m*_0_ and *m_cor_*_r_ is the mass of the sample (in grams) before corrosion and after corrosion days *τ*, *A* is the surface of the sample (in mm^2^) exposed to the corrosion determined at each stage of the corrosion test, and *ρ* is the alloy density—in this study, 1.74 g/cm^3^. 

### 3.7. Cytotoxicity

#### 3.7.1. Preparation of Material Extracts

The investigated wires were cut into pieces 1-cm long and then sterilized in 70% ethanol (water solution) followed by rinsing in phosphate-buffered saline (PBS). The sterilized materials were left under laminar flow for 24 h to dry. To study the cytotoxicity of the threads, material extracts were used. Briefly, following sterilization, the wire pieces were soaked for 72 h in a culture medium (1 mL/cm) consisting of Minimum Essential Medium α (MEM α, Thermo Fisher Scientific, Waltham, MA, USA) and 10% fetal bovine serum (FBS, Thermo Fisher Scientific). The ratio of surface area to extract volume, following ISO 10993-12: 2012 (E), was 3 cm^2^/mL (1X). Afterwards, the wires were washed in PBS and incubated in a fresh medium with antibiotics (1 mL medium/wire piece) for an additional 24 hours. Then, the extracts were harvested for later addition to the cells and diluted in culture medium to obtain the following ratios: 3 cm^2^/mL (1X), 1.5 cm^2^/mL (0.5X), 0.75 cm^2^/mL (0.25X). 

#### 3.7.2. Cell Cultures

Human periodontal ligament (hPDL) cells were obtained, according to the method of Bakkar [39]. All experiments with hPDL cells were performed in accordance with the Guidelines of “Directive 2004/23/EU of the European Parliament and of the Council” and they were approved by the local bioethics committee at the Jagiellonian University in Kraków, Poland, Dec. No. 1072.6120.253.2017. Both hPDL and human bone osteosarcoma SaOS-2 cells (SigmaAldrich) were expanded in a growth medium consisting of Minimum Essential Medium α (MEM α), 10% fetal bovine serum (FBS) from ThermoFisher Scientific and antibiotics (ZellShield®). Human PDL and SaOS-2 cells were seeded at the density of 2 × 10^4^ cells/well in 24-well plates in a culture medium composed of MEM α, 10% FBS, and 1% antibiotics. After 24-h initial cell culture, the culture media were removed, cells were washed with PBS and the extracts from studied materials were distributed to three culture wells using the extract ratio wire-processing method. The cells cultured on tissue-culture plates (TCP) without extracts were used as a general reference. Following the 24-h culture with the extracts, cells were washed with PBS and each culture well was covered with 200 μL of MTS reagent (CellTiter96Aqueous One Solution Cell Proliferation Assay; Promega) diluted 10× in a phenol-free MEM α. For the colorimetric reaction development, cells were incubated in the culture incubator until an obvious colour change of the MTS solution, from yellow to brownish, was observed. The solution was then transferred to individual wells in 96-well plate and the absorbance at 492 nm was read using a plate reader. The results were expressed as % cell viability vs. TCP control.

#### 3.7.3. Statistical Analyses

All biological data were collected in triplicate and expressed as mean ± SD. Statistical analyses were performed using one-way ANOVA and Bonferroni multiple comparisons test to calculate statistically significant differences at the *p*-value less than 0.05.

## 4. Numerical Results

### 4.1. Extrusion Simulation

**Figure 3:**
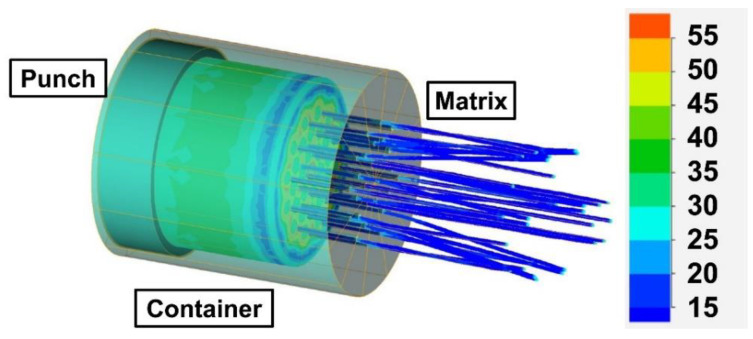
A three-dimensional model of the extrusion and flow stress distribution for the extrusion velocity of 0.25 mm/s and initial temperature of workpiece and matrix of 400 °C.

**Figure 4:**
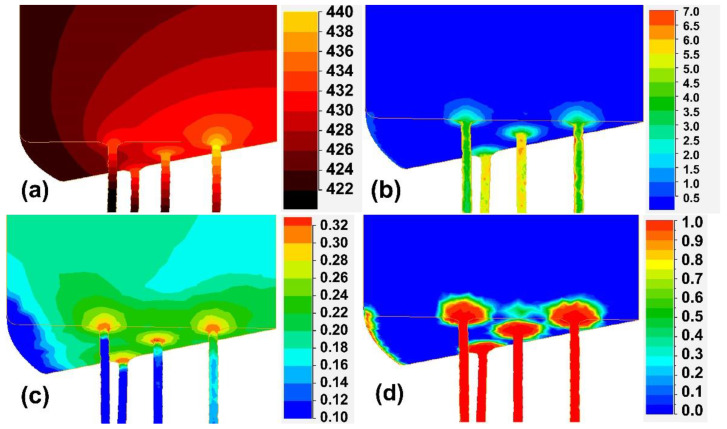
An exemplary result of the simulation, the extrusion velocity of 0.25 mm/s, the initial billet temperature equal to 400 °C: the distribution of temperature (**a**), effective plastic true strain (**b**), critical deformation, calculated by Equation (5) (**c**), and the dynamically recrystallized volume fraction (**d**).

In the FEM model of the extrusion, the shape and dimensions of the matrix and the workpiece corresponded to those that used in the real experiment. The full geometric model of the process with the distribution of the flow stress in the workpiece from the side of the matrix is shown in Figure 3. Due to the symmetry, the geometrical model used for the calculation was limited to 1/12 volume of the system. Exemplary results for the initial temperature of the matrix and the workpiece of 400 °C and the extrusion velocity of 0.25 mm/s (which corresponded, exactly, to the experimental condition) are shown in Figure 4. One can see that, in the steady-state phase of extrusion, the material temperature rose to 430–440 °C (Figure 4a) and the material’s true strain was in the range of 5.0–7.0 (Figure 4b), while the maximum critical strain, calculated by Equation (5), was not higher than 0.32 (Figure 4c). Thus, one can expect that the extruded material should be completely dynamically recrystallized (Figure 4d). 

### 4.2. Hot Drawing Simulation

The temperature and recrystallization fraction distribution during the multi-pass hot-drawing process were simulated by a Qform FE program. The modelled process also exactly corresponded to that used in the real experiment (Figure 1). Under such conditions, heat exchange between the furnace, die, and the wire occurs mainly by convection. According to the database of the Qform software, the heat-exchange coefficient between the die and the wire should be 2000 W/m^2^K, and between the air and the wire should be 120 W/m^2^K. Heat diffusion concerns energy transfer in the wire volume. An axisymmetric FE model was used for simulation. 

**Figure 5:**
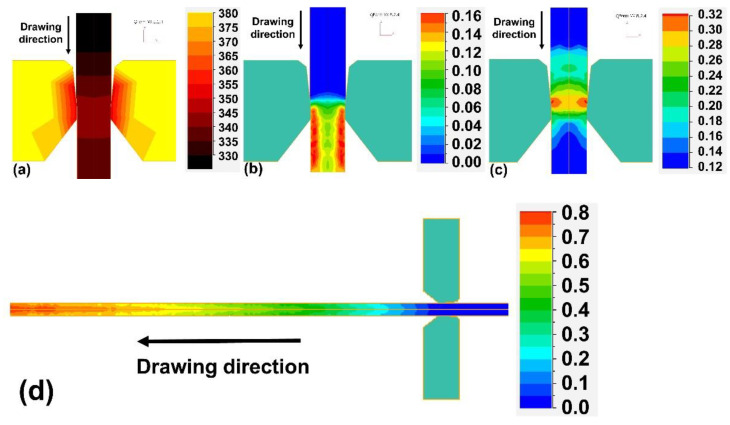
Results of the FE simulation of hot-drawing variant t440: the distribution of temperature in the deformation zone and hot die (**a**); effective plastic true strain (**b**); critical deformation, calculated by Equation (5) (**c**); and the static recrystallized volume fraction after the pass (**d**). Data for the first pass from Table 1.

An example of the simulation results is presented in Figure 5 for variants t440. According to these results, the critical strain calculated by Equation (5) should not be higher than 0.32 (Figure 5c) and the maximum true strain during a pass should be about 0.16 (Figure 5b). For the first pass from Table 1, the true strain at the wire surface and in its centre were predicted as 0.16 and 0.11, respectively (Figure 5b). It has been found that some non-homogenates in the strain distribution over a wire’s volume depends, insignificantly, both on the pass number from Table 1 and on the process’s temperature conditions. However, such distribution of strain can result in heterogenous grain size in the wire cross-section, which could be important for corrosion. Thus, in a further study, all corrosion tests and the microstructure and texture examinations were performed at the wires’ surfaces.

A three-passes hot-drawing process (see Table 1) was modelled for different profiles of the temperature. Based on the simulation results, it can be expected that the modelled material should be drawn in the increased temperature region and then relatively quickly cooled to the room temperature, due to the small diameter of the wire and its winding into a coil with large heat capacity. Directly before the next pass-through, the die was again heated in the furnace. According to the simulation results for variants t350, t400 and t440, the temperatures of these materials in their deformation zones were expected to be 280 °C, 310 °C, and 350 °C, respectively, which correspond to their homologous ones of 0.68, 0.71, and 0.87, also respectively. The predicted wire temperatures in the front of the die were slightly lower. After the pass and for the strain of 0.1, the predicted fractions of the recrystallized were 0.32, 0.55, and 0.80, respectively. Before the next pass, static recrystallization continued in the heating device, and the recrystallization amounts were predicted to be 0.8, 1.0, and 1.0 for the t350, t400, t440 temperature distributions, respectively. Thus, before the deformation, in all passes, complete recrystallization of the material was predicted for the t400 and t440 temperature distributions, and only partial recrystallization was predicted for the t350 temperature distribution. The results obtained also show that the calculated temperatures and the recrystallization amounts were approximately the same for all investigated passes. The slight difference may be related to the difference in each pass’s true strain and in the diameters of the passed wires (see Table 1). For this reason, we present the calculation results for the first pass only. The results of the simulations are summarized in Table 2.

## 5. The Experimental Results

### 5.1. Mechanical Properties

The tensile and bend tests results are collected in Table 3. It can be seen that the strengths of the wires, after the hot drawing, were significantly higher than the strength of the workpiece after the extrusion. An opposite pattern was observed for the A200 elongation and the number of bends required for it to crack (briefly, its bends number). Thus, the A200 elongation of the workpiece was higher than that of the wire after hot drawing. It was also noticed that increasing the hot-drawing temperature decreases the wire strength, from 263 MPa (variant t350) to 228 MPa (variant t440), and increased the maximal tensile elongation and the bends number, from 6.6% to 18.7% and from 2.3 to 6.0, respectively. The bends number was the highest for the wire drawn following the t440 mode.

### 5.2. In-Vitro Corrosion in a Solution Simulating Mammalian Body Fluid

**Figure 6:**
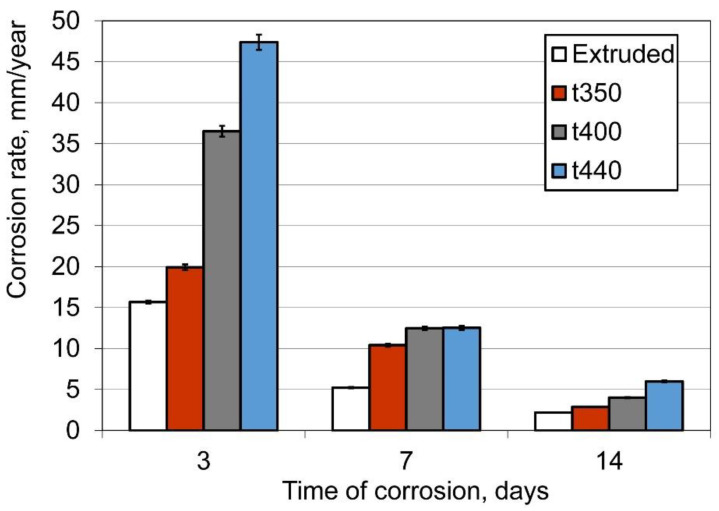
The effects of drawing temperature and the corrosion time on corrosion rate.

In addition to satisfactory mechanical properties, adequate corrosion characteristics are a key factor in the use of a wire as a BSW. Due to ethics and legal conditions, in-vivo study should be preceded by positive results in vitro. The first of such tests concerns the rate of corrosion, and our results are shown in Figure 6. About a 30–70% decrease in corrosion rate from decreases in drawing temperature was observed.

**Figure 7:**
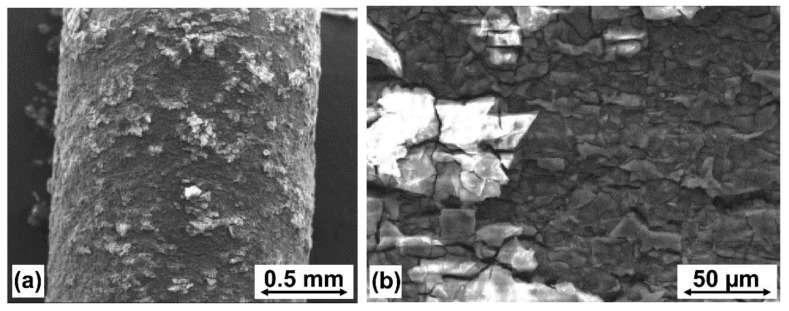
Typical images of the wire surface after 7 days of corrosion before cleaning (MgCa0.7 wire after extrusion, registered by scanning electron microscope). Wire surface in scale: (**a**) 0.5 mm; (**b**) 50 µm.

Typical images of the corroded wire surface, showing cracked corrosion products, were collected, and an example is shown in Figure 7. The image was registered during scanning electron microscopy study, using an S-3500N instrument from Hitachi (Hitachi Ltd., Tokyo, Japan). Similar images were recorded by [40] for the AZ80 magnesium alloy after exposure to body fluids-simulating chemicals.

### 5.3. Microstructure

**Figure 8:**
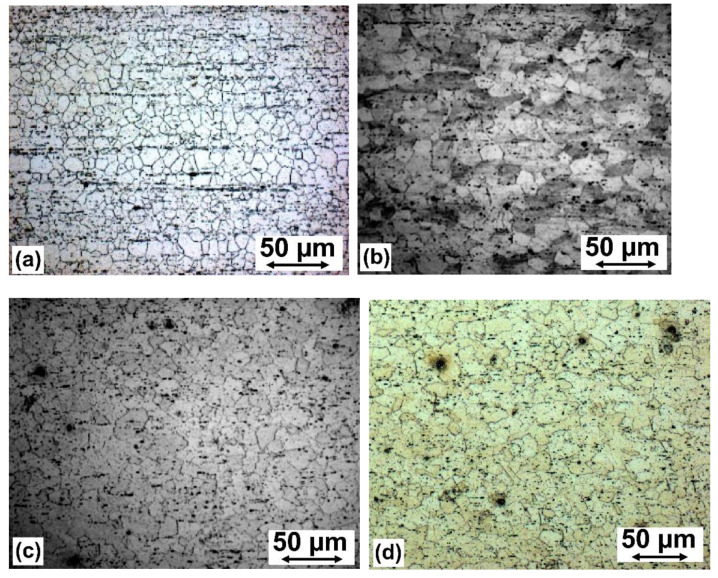
A typical microstructure of the hot-deformed material; as extruded (**a**) and extruded and hot-drawn for the temperature distribution t350 (**b**), t400 (**c**), and t440 (**d**). Longitudinal sections of the wires. The plastic flow direction along the horizontal axis.

Typical microstructures of the wires are shown in Figure 8. In these pictures, the drawing or extrusion direction is always along the horizontal axis. A completely recrystallized microstructure with equiaxed grains was typical for the as-extruded material (Figure 8a). This result confirms the computer simulation prediction that this material should be completely recrystallized (Figure 4d, Table 2). 

The drawn materials’ microstructures were also consistent with the FEM model predictions, as shown in Table 2. Some grains, elongated in the drawing direction, were observed in the microstructure of the material drawn at the t350 temperature distribution (Figure 8b) and may be related to incomplete recrystallization, as was expected from the simulations (Table 2). Equiaxed grains, typical for the temperature distributions t400 and t440—with slightly larger grains for the second of these variants—indicate complete recrystallization, also expected, based on the computer modelling results. 

**Figure 9:**
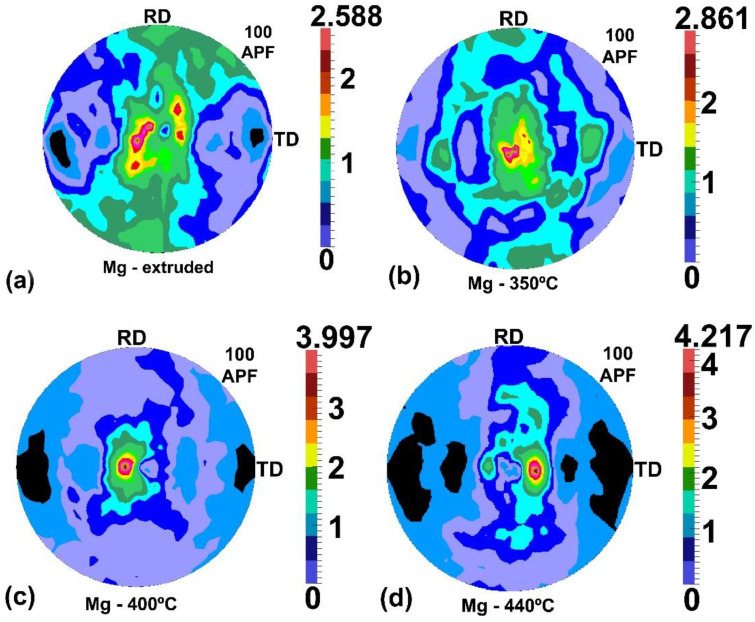
The complete pole figures (10.0) for the as-extruded material (**a**) and for the material extruded and drawn at t350 (**b**), t400 (**c**), and t440 (**d**).

During the X-ray diffraction study, peaks from phases with a hexagonal close-packed structure (*A3*, Pearson symbol *hP2*, space group no 194) were registered on XRD patterns. The dominant of these was identified as magnesium with the lattice parameters *a* = *b* = 3.2092(3) A, *c* = 5.2114(1) A, *α* = *β* = 90 °C, *γ* = 120 °C. The lattice parameters were very close to those for pure magnesium, which indicated low content of Mg-based elements in the solid solution. The second phase was identified as CaMg_2,_ with lattice parameters *a* = *b* = 6.2528 A, *c* = 10.1435 A, *α* = *β* = 90°, *γ* = 120°; and its amount was very low (on the order of tenths of a percent, i.e., 0.1%, according to results of the Rietveld analysis [41,42], LHPM codes [43] implemented in the HighScore Plus software v. 3.0e (3.0.5) by PANalytical B.V., Alemo, The Netherlands). No significant effect of the material state on CaMg_2_ amounts was found. The Ca content in the alloy was relatively low, so the above results confirmed the relatively low solubility of Ca in Mg. The average size of the CaMg_2_ particles was determined to be 1 μm. The magnesium-phase texture confirmed that the material had recrystallized (Figure 9). For the as-extruded material, the ODF maximum was of 9.1. For the extruded-and-drawn materials, this value increased with drawing temperature to 7.1, 8.2, and 17.9 for t350, t400, and t440, respectively. The same trend was displayed in the intensity of (10.0) those planes nearly parallel to the long axis of the wire. Thus, the maximum values (10.0) of the pole figures were 2.6, 2.9, 4.0, and 4.2 for the as-extruded and extruded-and-drawn materials at t350, t400, and t440, respectively. For the other pole figures, the values were usually lower (e.g., for the sample drawn at t440, the maximum value of the pole figures (11.0), (10.1), and (11.1) was 2.5, 2.4, and 2.0, respectively). Thus, only (10.0) pole figures are shown in Figure 9.

### 5.4. In-Vitro Cytotoxicity

**Figure 10:**
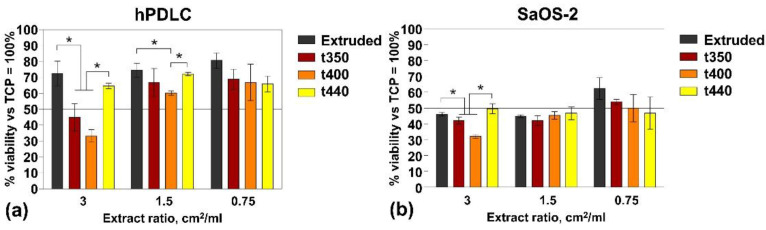
Viability of normal human PDL cells (**a**) and human bone osteosarcoma cell line SaOS-2 (**b**) cells exposed to different extracts ratios, harvested from MgCa alloys subjected to different modes of processing. * *p* < 0.05 compared between samples.

**Figure 11:**
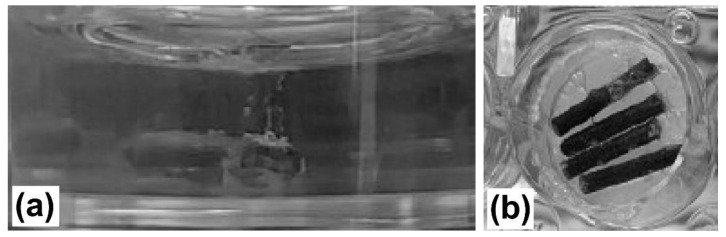
Visualization of extracts during materials incubation in culture medium: hydrogen generation (**a**), dark colour of wires surface after 72-h preincubation in culture media and change of media colour (**b**).

The cell viability results are shown in Figure 10, separately, for the hPDL and SaOS-2 cells. During incubation of the wires for the extracts’ preparation, hydrogen evolution was observed (Figure 11). After preincubation, the surfaces of the samples turned dark, and the extracts changed colour to purplish, which indicated that the pH of the medium had become alkaline.

Regarding normal hPDL cells, the cytotoxic effects was observed mostly for the 3 cm^2^/mL extract ratio, with the strongest effects observed obtained from the t400 and t350 wire-processing methods. A similar trend was observed with the 1.5 cm^2^/mL extract ratio, but the cells’ survival under the t400- and t350-processed wires increased significantly, by 26.79% and 21.94%, respectively. The addition of the 0.75 cm^2^/mL extracts to the culture medium did not show any dependencies of cell viability on wire-processing method, and no tested extracts decreased cell viability beyond 65.87%. Notably, the extracts from the t440-processed wire least influenced cell viability, regardless of the studied extract ratio. In contrast, the extracts had much stronger cytotoxic effects on the human bone osteosarcoma cell line SaOS-2, and both the 3 and 1.5 cm^2^/mL extract ratios decreased cell viability below 50%. In these cultures, the dependence of cells’ survival on the wire-processing method was observed only for the 3 cm^2^/mL extracts and the trend was similar to that observed in hPDL cell cultures. Further dilution of the extracts to 0.75 cm^2^/mL did not much improve the SaOS-2 cells’ survival, and their viability remained at the averaged level of 50.16% for all processed wires. 

## 6. Discussion

In the present study we confirmed the qualitative relationship between recrystallization amount and deformation condition, as predicted by FEM (i.e., microstructure and the mechanical properties). Quantitative determination of these dependencies is difficult due to the mutual influence of recrystallization and precipitates, especially given that the distributions of particles on the cross-sections of the extruded samples were not uniform (Figure 8a). Moreover, as the Mg-Ca eutectic temperature is only about 516 °C, the solid solubility of Ca in Mg is relatively low (i.e., about 0.9 wt. % at the eutectic temperature and 0.1 wt.% at the 350 °C [44]) and the corresponding part of the phase-equilibrium diagram has not been precisely determined in the available literature [45,46]. 

We have not found any effect of an alloy’s microstructure on its corrosion characteristic. According to our results, shown in Figure 6, an apparent increase in the corrosion rate due to drawing can be seen, and both the drawing temperature and the time of corrosion (*τ*) affected the corrosion rate (*δ*) of hot-drawn wires. The effect of the deformation temperature (*t*) (i.e., the material temperature in the deformation zone) on the corrosion rate can be expressed as:

(9) $$\delta =\delta_{0}+k\;exp\left(\alpha \tau \right)\;exp\left(\beta t\right),$$

where: *δ*_0_, *k*, *α*, *β* are empirical coefficients, fitted by the least-squares method, of value *k* = 1.7905; *α* = -0.35849; *β* = 0.012447; *δ*_0_ = 4.4143. The average error of the approximation for the data from Figure 6 was equal to 7%.

The revealed decrease in the corrosion rate associated with a reduction in the drawing temperature and over the duration of the corrosion test may be related to the differences in the surface layer formed during corrosion at elevated temperature, and also to some subtle differences in microstructure. Furthermore, the above may also be related to discrete differences in the distribution of grain size and particles and in the grain boundaries’ chemistry. Some reports from the literature suggest that the surface film formed on magnesium alloys in the air may have protective properties, resulting in an anti-corrosive effect in mild or weakly corrosive environments—at least during the early stages of corrosion [35]; usually this applies to the first three days of corrosion [47]. The present study examined time periods of 3 to 14 days. So, further detailed study is required to clarify the effect observed by us. 

The c/a ratio of the magnesium lattice cell (equal to 1.624) is very close to that of the ideal hcp structure (i.e., equal to 1.633) and <10.0>-type texture components developed in magnesium during extrusion or drawing at temperatures below 450 °C [48]. The same type of a strong-fibre texture developed during the hot extrusion of the magnesium alloy AZ31 [49]. The component <10.0> is typical for cold-drawn and/or extrused textures of metals with *c*/*a* < 1.633 (i.e., such as Zr, Ti and Be). However, in the case of magnesium, additional texture components can develop. According to [50] <10.0>–<11.0>-type fibre textures develop in wires of magnesium alloy AZ30 during static recrystallization (see Figure 8c in [50]). The same type of the two-component texture developed in pure magnesium during cold drawing at room temperature, including even high deformations, as reported in [51]. As for hcp structure, the directions <10.0> and <11.0> are perpendicular to the planes (10.0) and (11.0), respectively. Thus, one can conclude that the results of the present study are consistent with the literature. However, it should be the noted that, in the literature, measurements of textures have usually been made on wires’ cross-sections; therefore, surface textures are typically determined differently than was done in our present research.

**Figure 12:**
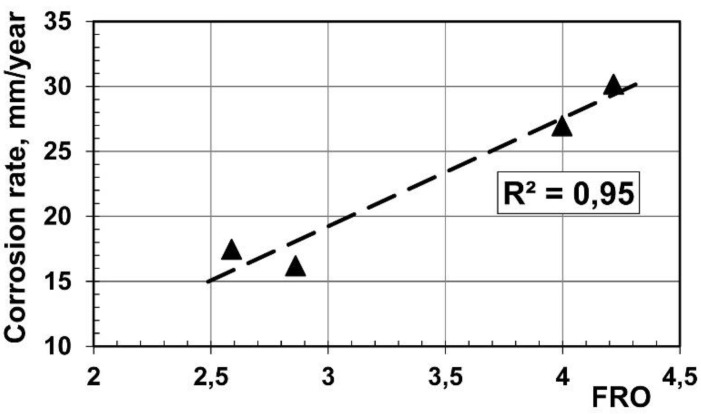
A correlation between the (10.0) pole figure’s maximum and the rate of corrosion.

A fairly good correlation between the (10.0)-pole figure’s maximum and the rate of corrosion was observed in the present study (Figure 12). Such good correlation was not found for other components of the texture (e.g., the correlation coefficient for the (11.0)-pole figure’s maximum was below 0.1). The effect of crystal orientation on corrosion was previously reported in the literature [52,53,54]. The texture effect on the biocorrosion of the magnesium-based, AZ80, has also been noted in the literature (e.g., [40]). Work [55] reported relatively high corrosion resistance of the orientation (00.2) by comparison with the corrosion test results obtained for a material rolled and then 2.5-h annealed at 340 °C and another, additionally compressed, to a strain of 9%. However, such treatments changed both the textures and microstructures of the tested materials. The plastic deformation effect on corrosion is well known [56], including for magnesium alloys [57]. It is plausible that the deformation effect probably insignificantly influenced the result of [55] because of similar conclusions drawn from biocorrosion studies of magnesium single crystals [58]. Although the practical possibilities of texture changes during the hot drawing of magnesium are limited, they can be applied, to some extent, to influence the corrosion resistance, as has been shown in the present study.

The results of the cytotoxicity study indicated that the toxicity of the material dissolution products (i.e. material extracts) (Figure 10) cannot be directly predicted from the drawing temperatures or corrosion rates of the studied wires (Figure 6). The latter can be partly explained by the fact that the release of an excessive amounts of Mg^2+^ ions due to corrosion is associated with the formation of Mg(OH)_2_ [59], and this may affect pH changes in the extracts, leaving them alkaline. It has already been reported that an alkaline environment can inhibit the growth of gastric cancer [60] or human melanoma cells [61]. Taking into account the intended clinical application of MgCa0.7 alloys, pH changes may not be the only beneficial effect, as seen in vivo; hydrogen gas has also antitumor properties [62] and an ability to reduce inflammation and oxidative stress, in vivo [63], while the delivery of Mg^2+^ promotes a microenvironment that enhances bone regeneration [64].

Some important patterns, however, were noted, especially when using the 3cm^2^/mL extracts ratio, as these studies consistently showed the t400 and t350 processing methods as the harshest to cell viability, yet the extruded rods were the least toxic material (Figure 10). This may be due to lower corrosion rates vs. the processing of the materials and some differences in their textures, in comparison with the material after drawing. The influence of differences in recrystallization amounts is also possible. Overall, the material obtained at the maximum drawing temperature (mode t440) seem the most optimum for biological applications, as the extracts from these materials, at any studied concentration, affected cell viability the least.

The cytotoxicity study also provided new insights into the potential anti-cancer activity of the wires. The largest average absolute difference in the viability of healthy and cancer cells for studied extract ratios was observed for the material after extrusion (23.3%) and after hot drawing at the maximum temperature (21.2%). We believe this finding is of great importance for future biomedical applications and certainly worth of more extensive research regarding such anti-cancer activity.

We cannot state, at this point in our research, that the death of cancer cells was associated with the high level of media alkalization for the t440 wires, as the extracts from these wires did not much affect the growth of normal hPDL. Moreover, the extruded wires displayed the lowest corrosion rates, despite their extracts being toxic to SaOS-2 cells and non-toxic to hPDL. Thus, it seems plausible that some components of these extracts selectively inhibit the growth of cancer cells without affecting normal human cells, and this is not a simple consequence of media alkalization.

## 7. Conclusions 

The manufacturing technology of a biodegradable wire of MgCa0.7 alloy was successfully developed as a result of the present study. This wire can be anticipated as a potential candidate for some applications in surgery. Its mechanical properties and corrosion characteristics, determined in this study, justify taking this material into consideration as a candidate for further, more extensive biological studies required for its clinical application.

Some influence of the metal-forming parameters on the product’s mechanical properties, and of its subsurface texture on its bio-corrosion in simulated body fluids, were found in the present study. It was also shown that a compromise is required for the choice of the optimal hot-drawing parameters, enabling the manufacture of high-strength, biodegradable surgical wires. A higher drawing temperature from the process-parameters window resulted in a product of increased ductility and rate of in-vitro corrosion in a bovine serum and decreased strength. 

The cytotoxicity study showed that the temperature used in hot drawing significantly affects cell viability. Moreover, all studied materials were more toxic to cancer cells than to healthy ones. This difference in cytotoxicity partly depended on the processing parameters and it was maximal, for rods, after extrusion, and, for wires, by hot drawing at 440 °C. Thus, the selected processing parameters make it possible to obtain a product that is not only a promising candidate for future biodegradable surgical wire, but it also displays intrinsic bioactive properties, specifically, anti-tumor activity. 

Definitive optimal hot-drawing parameters requires further study, including the examination of wires in different biological fluids and assessment of their specific properties, such as knot strength and relaxation, followed by more detailed in-vitro and in-vivo biological examination.

## Figures and Tables

**Table 1:** Drawing schedule.

No. of the Pass	1	2	3
Diameter of the wire after pass, mm	1.72	1.64	1.56
True strain in the pass	0.091	0.095	0.101

**Table 2:** The results of FEM simulation.

Process	Temperature in Deformation Zone, °C	Fraction of the Recrystallized Material after Pass	Fraction of the Recrystallized Material before Next Pass
Extruded	430–440	1.0	-
t350	280	0.32	0.8
t400	310	0.55	1.0
t440	350	0.8	1.0

**Table 3:** Mechanical properties of the material after extrusion and hot drawing.

	Rp, MPa	Rm, MPa	A_200_, %	Number of Bends
Extruded	126.3 ± 2.5	210.3 ± 1.16	26.7 ± 1.5	4.0 ± 0.50
t350	221.7 ± 8.07	263.0 ± 2.26	6.6 ± 1.92	2.3 ± 0.57
t400	188.0 ± 5.0	247.0 ± 6.45	15.1 ± 2.35	2.6 ± 0.56
t440	154.0 ± 6.4	228.0 ± 0.9	18.7 ± 1.79	6.0 ± 0.81

## Data Availability

Data is contained with the article.

## References

[1] Seitz J.-M., Durisin M., Goldman J., Drelich J.W. (2015). Recent Advances in Biodegradable Metals for Medical Sutures: A Critical Review. Adv. Healthc. Mater..

[2] Seitz J.-M., Eifler R., Bach F.-W., Maier H.J. (2014). Magnesium degradation products: Effects on tissue and human metabolism. J. Biomed. Mater. Res. A.

[3] Schümann K., Ettle T., Szegner B., Elsenhans B., Solomons N.W. (2007). On risks and benefits of iron supplementation recommendations for iron intake revisited. J. Trace Elem. Med. Biol..

[4] Nriagu J. (2019). Encyclopedia of Environmental Health.

[5] Witte F. (2010). The history of biodegradable magnesium implants: A review. Acta Biomater..

[6] Zakiyuddin A., Lee K. (2015). Effect of a small addition of zinc and manganese to Mg–Ca based alloys on degradation behavior in physiological media. J. Alloys Compd..

[7] Seelig M.G. (1924). A study of magnesium wire as an absorbable suture and ligature material. Arch. Surg..

[8] Lambotte A. (1932). L’utilisation du magnésium comme matériel perdu dans l’ostéosynthèse. Bull MémSoc Nat Cir..

[9] Seitz J.-M., Utermöhlen D., Wulf E., Klose C., Bach F.-W. (2011). The Manufacture of Resorbable Suture Material from Magnesium—Drawing and Stranding of Thin Wires. Adv. Eng. Mater..

[10] Hassel T., Bach F.W., Golovko A., Krause C. (2006). Investigation of the mechanical properties and the corrosion behaviour of low alloyed magnesium-calcium alloys for use as absorbable biomaterial in the implant technique. 45th Annu. Conf. Metall. CIM.

[11] Chen Y.-T., Hung F.-Y., Syu J.-C. (2019). Biodegradable Implantation Material: Mechanical Properties and Surface Corrosion Mechanism of Mg-1Ca-0.5Zr Alloy. Metals.

[12] Xue J., Griebel A.J., Zhang Y., Romany C., Chen B., Schaffer J., Weihs T.P. (2021). Influence of Thermal Processing on Resoloy Wire Microstructure and Properties. Adv. Eng. Mater..

[13] Antoniac I., Adam R., Bit A., Miculescu M., Trante O., Petrescu I.M., Pogarasteanu M. (2021). Comparative Assessment of In Vitro and In Vivo Biodegradation of Mg-1Ca Magnesium Alloys for Orthopedic Applications. Materials.

[14] Hou R., Victoria-Hernandez J., Jiang P., Willumeit-Römer R., Luthringer-Feyerabend B., Yi S., Letzig D., Feyerabend F. (2019). In vitro evaluation of the ZX11 magnesium alloy as potential bone plate: Degradability and mechanical integrity. Acta Biomater..

[15] Seitz J.-M., Eifler R., Stahl J., Kietzmann M., Bach F.-W. (2012). Characterization of MgNd2 alloy for potential applications in bioresorbable implantable devices. Acta Biomater..

[16] Wan Y., Xiong G., Luo H., He F., Huang Y., Zhou X. (2008). Preparation and characterization of a new biomedical magnesium–calcium alloy. Mater. Des..

[17] Milenin A., Kustra P., Byrska-Wójcik D., Wróbel M., Paćko M., Sulej-Chojnacka J., Matuszyńska S., Płonka B. (2020). The effect of in vitro corrosion on the mechanical properties of metallic high strength biodegradable surgical threads. Arch. Civ. Mech. Eng..

[18] Milenin A., Kustra P., Byrska-Wójcik D., Wróbel M., Packo M., Sulej-Chojnacka J., Matuszynska S., Pidvysots’Kyy V. The influence of the parameters of hot drawing of MgCa alloys wires on the mechanical properties that determine the applicability of the material as a high strength biodegradable surgical thread. Proceedings of the Procedia Manufacturing.

[19] Milenin A., Kustra P., Byrska-Wójcik D., Grydin O., Schaper M., Mentlein T., Gerstein G., Nürnberger F. (2016). Analysis of microstructure and damage evolution in ultra-thin wires of the magnesium alloy MgCa0.8 at multipass drawing. JOM.

[20] Kustra P., Milenin A., Byrska-Wójcik D., Grydin O., Schaper M. (2017). The process of ultra-fine wire drawing for magnesium alloy with the guaranteed restoration of ductility between passes. J. Mater. Process. Technol..

[21] Aung N.N., Zhou W. (2002). Effect of heat treatment on corrosion and electrochemical behaviour of AZ91D magnesium alloy. J. Appl. Electrochem..

[22] Aung N.N., Zhou W. (2010). Effect of grain size and twins on corrosion behaviour of AZ31B magnesium alloy. Corros. Sci..

[23] Liu M., Qiu D., Zhao M.-C., Song G., Atrens A. (2008). The effect of crystallographic orientation on the active corrosion of pure magnesium. Scr. Mater..

[24] Zhu S., Liu Z., Qu R., Wang L., Li Q., Guan S. (2013). Effect of rare earth and Mn elements on the corrosion behavior of extruded AZ61 system in 3.5 wt% NaCl solution and salt spray test. J. Magnes. Alloy..

[25] Anisimova N., Kiselevskiy M., Martynenko N., Straumal B., Willumeit-Römer R., Dobatkin S., Estrin Y. (2020). Cytotoxicity of biodegradable magnesium alloy WE43 to tumor cells in vitro: Bioresorbable implants with antitumor activity?. J. Biomed. Mater. Res. Part B Appl. Biomater..

[26] Fischer J., Pröfrock D., Hort N., Willumeit R., Feyerabend F. (2011). Improved cytotoxicity testing of magnesium materials. Mater. Sci. Eng. B Solid-State Mater. Adv. Technol..

[27] Kolmogorov N. (1937). Statistical theory of crystallization of metals. Izv. Akad. Nauk SSSR, Ser. Mat. Bull. Acad. Sci. USSR. Ser. Math.

[28] Johnson W.A., Mehl R.F. (1939). Reaction Kinetics in Processes of Nucleation and Growth. Trans. Am. Inst. Min. Metall. Eng..

[29] Avrami M. (1939). Kinetics of Phase Change. I General Theory. J. Chem. Phys..

[30] Sellars C.M. (1979). Physical Metallurgy of Hot Working. Proceedings of the International Conference of Hot Working and Forming Processes.

[31] Milenin A., Kustra P., Pietrzyk M. (2014). Physical and numerical modelling of wire drawing process of Mg alloys in heated dies accounting for recrystallization. Key Eng. Mater..

[32] Biba N., Maximov A., Stebunov S., Vlasov A. The model for simulation of thermally, mechanically and physically coupled problems of metal forming. Proceedings of the 14th International conference on Metal Forming.

[33] Friedrich H.E., Mordike B.L. (2006). Magnesium Technology.

[34] Milenin A., Kustra P., Wróbel M., Paćko M., Byrska-Wójcik D. (2019). Comparison of the stress relaxation of biodegradable surgical threads made of Mg and Zn alloys and some commercial synthetic materials. Arch. Metall. Mater..

[35] Atrens A., Song G.-L., Liu M., Shi Z., Cao F., Dargusch M.S. (2015). Review of recent developments in the field of magnesium corrosion. Adv. Eng. Mater..

[36] Schulz L.G. (1949). A Direct Method of Determining Preferred Orientation of a Flat Reflection Sample Using a Geiger Counter X-Ray Spectrometer. J. Appl. Phys..

[37] LaboTex, The Texture Analysis Software for Windows. http://www.labosoft.com.pl/.

[38] Biowest. https://www.biowest.net.

[39] Bakkar M., Liu Y., Fang D., Stegen C., Su X., Ramamoorthi M., Lin L.C., Kawasaki T., Makhoul N., Pham H. (2017). A simplified and systematic method to isolate, culture, and characterize multiple types of human dental stem cells from a single tooth. Methods in Molecular Biology.

[40] Xiong Y., Yang Z., Zhu T., Jiang Y. (2020). Effect of texture evolution on corrosion resistance of AZ80 magnesium alloy subjected to applied force in simulated body fluid. Mater. Res. Express.

[41] Rietveld H.M. (1967). Line profiles of neutron powder-diffraction peaks for structure refinement. Acta Cryst..

[42] Rietveld H.M. (1969). A profile refinement method for nuclear and magnetic structures. J. Appl. Cryst..

[43] Hill R.J., Howard C.J. (1986). A Computer Program for Rietveld Analysis of Fixed Wavelength X-ray and Neutron Diffraction Patterns.

[44] Crosby R.L., Fowler K.A. (1963). Effect of Indium on the Solid Solubility of Calcium and Silicon in Magnesium.

[45] Nayeb-Hashemi A.A., Clark J.B., Nayeb-Hashemi A.A., Clark J.B. (1988). Phase Diagrams of Binary Magnesium Alloys.

[46] Mezbahul-Islam M., Mostafa A.O., Medraj M. (2014). Essential Magnesium Alloys Binary Phase Diagrams and Their Thermochemical Data. J. Mater..

[47] Feliu S. (2019). Composition, Structure, and Protective Properties of Air-Formed Oxide Films on Magnesium Alloys. Magnesium and Its Alloys.

[48] Hu H. (1974). Texture of Metals. Texture.

[49] Fan X., Tang W., Zhang S., Li D., Peng Y. (2010). Effects of dynamic recrystallization in extruded and compressed AZ31 magnesium alloy. Acta Metall. Sin. (Engl. Lett.).

[50] Chao H.Y., Sun H.F., Chen W.Z., Wang E.D. (2011). Static recrystallization kinetics of a heavily cold drawn AZ31 magnesium alloy under annealing treatment. Mater. Charact..

[51] Chen X.M., Li L.T., Chen W., Zhang W.C., Zhang L.X., Qiao Y.D., Wang E.D. (2017). Fine-grained structure and recrystallization at ambient temperature for pure magnesium subjected to large cold plastic deformation. Mater. Sci. Eng. A.

[52] Szklarz Z., Wróbel M., Krawiec H. The influence of crystallographic orientation of grains on corrosion behavior of aluminum in sodium chloride solution. Proceedings of the 6th Kurt Schwabe Symposium: Surface analysis and material engineering in corrosion science and electrochemical technologies.

[53] Xin R., Luo Y., Zuo A., Gao J., Liu Q. (2012). Texture effect on corrosion behavior of AZ31 Mg alloy in simulated physiological environment. Mater. Lett..

[54] Zhu S.-J., Liu Q., Qian Y.-F., Sun B., Wang L.-G., Wu J.-M., Guan S.-K. (2014). Effect of different processings on mechanical property and corrosion behavior in simulated body fluid of Mg-Zn-Y-Nd alloy for cardiovascular stent application. Front. Mater. Sci..

[55] Xiong Y., Jiang Y. (2016). Cyclic deformation and fatigue of rolled AZ80 magnesium alloy along different material orientations. Mater. Sci. Eng. A.

[56] Dorlot J.M., Baïlon J.P. (2000). Des Materiaux.

[57] Snir Y., Ben-Hamu G., Eliezer D., Abramov E. (2012). Effect of compression deformation on the microstructure and corrosion behavior of magnesium alloys. J. Alloys Compd..

[58] Hagihara K., Okubo M., Yamasaki M., Nakano T. (2016). Crystal-orientation-dependent corrosion behaviour of single crystals of a pure Mg and Mg-Al and Mg-Cu solid solutions. Corros. Sci..

[59] Johnston S., Shi Z., Venezuela J., Wen C., Dargusch M.S., Atrens A. (2019). Investigating Mg Biocorrosion In Vitro: Lessons Learned and Recommendations. JOM.

[60] Li W., Zhou Y., Shang C., Sang H., Zhu H. (2020). Effects of environmental pH on the growth of gastric cancer cells. Gastroenterol. Res. Pract..

[61] Azzarito T., Lugini L., Spugnini E.P., Canese R., Gugliotta A., Fidanza S., Fais S. (2016). Effect of Modified Alkaline Supplementation on Syngenic Melanoma Growth in CB57/BL Mice. PLoS ONE.

[62] Li Q., Tanaka Y., Miwa N. (2017). Influence of hydrogen-occluding-silica on migration and apoptosis in human esophageal cells in vitro. Med. Gas Res..

[63] Zhou H., Han B., Hou L.-M., An T.-T., Jia G., Cheng Z.-X., Ma Y., Zhou Y.-N., Kong R., Wang S.-J. (2016). Protective Effects of Hydrogen Gas on Experimental Acute Pancreatitis. PLoS ONE.

[64] Grigolato R., Pizzi N., Brotto M.C., Corrocher G., Desando G., Grigolo B. (2015). Magnesium-enriched hydroxyapatite as bone filler in an ameloblastoma mandibular defect. Int. J. Clin. Exp. Med..

